# Defensive effect of microRNA-200b/c against amyloid-beta peptide-induced toxicity in Alzheimer's disease models

**DOI:** 10.1371/journal.pone.0196929

**Published:** 2018-05-08

**Authors:** Sayuri Higaki, Masashi Muramatsu, Akio Matsuda, Kenji Matsumoto, Jun-ichi Satoh, Makoto Michikawa, Shumpei Niida

**Affiliations:** 1 Medical Genome Center, National Center for Geriatrics and Gerontology, Obu, Aichi, Japan; 2 Division of Phenotype Disease Analysis, Institute of Resource Development and Analysis, Kumamoto University, Kumamoto, Japan; 3 Department of Allergy and Clinical Immunology, National Research Institute for Child Health and Development, Setagaya-ku, Tokyo, Japan; 4 Department of Bioinformatics and Molecular Neuropathology, Meiji Pharmaceutical University, Kiyose, Tokyo, Japan; 5 Department of Biochemistry, Nagoya City University Graduate School of Medical Sciences, Nagoya, Aichi, Japan; Universita degli Studi di Napoli Federico II, ITALY

## Abstract

MiRNA molecules are important post-transcriptional regulators of gene expression in the brain function. Altered miRNA profiles could represent a defensive response against the pathogenesis of neurodegenerative disorders, such as Alzheimer's disease (AD). Endogenous miRNAs have lower toxic effects than other gene silencing methods, thus enhancing the expression of defensive miRNA could be an effective therapy. However, little is known about the potential of targeting miRNAs for the treatment of AD. Here, we examined the function of the miR-200 family (miR-200a, -141, -429, -200b, -200c), identified using miRNA microarray analysis of cortical tissue from Tg2576 transgenic mice. In murine primary neurons, we found that upregulation of miR-200b or -200c was induced by the addition of amyloid beta (Aβ). Neurons transfected with miR-200b or -200c reduced secretion of Aβ in conditioned medium. Moreover, mice infused with miR-200b/c into the brain were relieved of memory impairments induced by intracerebroventricular injection of oligomeric Aβ, and demonstrated proper spatial learning in the Barnes maze. To gain further understanding of the relationship between miR-200b/c and Aβ, we identified target mRNAs via an RNA-binding protein immunoprecipitation-microarray assay. Western blot analysis showed that expression of ribosomal protein S6 kinase B1 (S6K1), a candidate target, was inhibited by miR-200c. S6K1, a downstream effector of mammalian target of rapamycin (mTOR), serves as a negative feedback mediator that phosphorylates insulin receptor substrate 1 at serine residues (IRS-1pSer). S6K1-dependent IRS-1pSer suppresses insulin signaling leading to insulin resistance, which is frequently observed in AD brains. Notably, miR-200b/c transfection of SH-SY5Y cells reduced the levels of IRS-1pSer. This finding indicates that miR-200b/c has the potential to alleviate insulin resistance via modulation of S6K1. Taken together, miR-200b/c may contribute to reduce Aβ secretion and Aβ-induced cognitive impairment by promoting insulin signaling.

## Introduction

Alzheimer’s disease (AD) is an age-related neurodegenerative disease that is currently the most common cause of dementia worldwide, and is characterized by the hallmark pathology of amyloid-beta (Aβ) deposition [1]. Accumulating evidence supports the amyloid hypothesis of AD pathogenesis, i.e., that Aβ peptides play a crucial role in initiating the disease and trigger a complex pathological cascade, which leads to neuronal damage [2]. Aβ peptide is derived from amyloid precursor protein (APP) by sequential proteolytic processing by α-, β-site amyloid precursor protein-cleaving enzyme 1 (BACE1) and γ-secretase [3]. Excessive accumulation and secretion of Aβ, especially Aβ1–42, which is cleaved by BACE1 and γ-secretase, may facilitate the aggregation of senile plaques in the brains of elderly individuals, leading to increased neurodegeneration [4].

The mouse model of AD, Tg2576, expresses the Swedish mutant of APP under control of the hamster prion protein promoter [5]. In the Tg2576 mouse brain, amyloid plaque deposition reaches detectable levels around 9–10 months, and dense Aβ plaques are most frequently observed in the cortex, subiculum, and presubiculum [5]. Between 15 and 23 months of age, Aβ plaques in the Tg2576 mouse brain accumulate to levels similar to those seen in the brains of patients with AD [6]. Abnormal amyloid assembly and deposition can disrupt normal neuronal activity. Behavioral assessments have shown that Tg2576 mice exhibit spatial memory decline at as early as 10 months of age [7, 8]. Accumulation of Aβ is not sufficient to explain the pathology of AD, although it certainly induces some of the characteristic symptoms.

Over the past decade, epidemiological observations have supported a connection between AD and type 2 diabetes. Impaired insulin signaling is thought to be involved in AD [9, 10]. AD pathology is sometimes referred to as “type 3 diabetes”, because postmortem AD brains present poor responsiveness to insulin, that is, insulin resistance, independent of peripheral insulin levels [11]. Recent evidences suggest that insulin signaling has important role to be neuroprotective [12, 13], and to control synapse formation [14]. Oligomeric Aβ leads to loss of insulin receptors from synaptic plasma membranes [13, 15], and inhibition of neural insulin receptor substrate 1 (IRS-1) [16]. Insulin resistance can induce autophagosome accumulation, which contributes to additional Aβ peptide generation [17]. Thus, unfavorable interactions between insulin signaling and Aβ may ultimately result in memory impairment.

MicroRNAs (miRNAs) are small, non-coding, regulatory RNA molecules that fine-tune cellular gene expression at a post-transcriptional level. They are predicted to regulate about 30% of protein-coding genes and up to 90% of the genome. Importantly, approximately 70% of all identified miRNAs are expressed in the brain [18]. Disease can disrupt their coordinated expression; thus, changes in miRNA expression may result in pathogenesis.

MiRNAs also have a therapeutic role. MiRNA based-RNA interference (RNAi) therapies have an advantage in that they have lower toxicity than other gene-silencing methods, such as short hairpin RNAs [19]. Delivery of miRNAs *in vivo* successfully suppresses tumor activities without inducing toxicity [20]. Intravenous administration of nanoparticles, including anti-miRNA, decreases the cell proliferation and tumor size of glioblastomas in the brain as well as in peripheral organs [21]. However, to date most studies have focused on the role of miRNAs in tumor suppression, with the development of miRNA therapy for AD lagging behind that in cancer research.

In the present study, we aimed to identify differential expression of miRNAs in the early period of Aβ accumulation in the Tg2576 mouse brain, when the brain is naive to Aβ toxicity, in order to avoid the complicating effects of inflammation and dysregulation that follows long-term exposure to Aβ. Then, the effectiveness of potential therapeutic miRNAs were validated *in vitro* and *in vivo*. We further investigated the target genes and signaling of therapeutic miRNAs aimed at alleviating against Aβ-induced toxicity.

## Material and methods

### Animals

Wild-type C57BL/6J mice (CLEA Japan Inc, Kawasaki, Japan) and Tg2576 mice (Taconic Biosciences Inc., Hudson, NY, USA) at 10- and 17-months-old were used for RNA extraction from brain cortex tissue. For the isolation of primary murine neuronal cells, pregnant C57BL/6J mice (embryonic-day 17) were euthanized and the embryos were collected. For behavioral testing, male C57BL/6J mice (13–14-weeks-old) were housed in plastic cages and kept in a regulated environment with a 12-h light/dark cycle. Food and tap water were available *ad libitum*.

All animal studies were performed according to the protocols approved by the Ethics Committees of the National Center for Geriatrics and Gerontology, Japan. The procedures involving mice and their care were conducted in line with international guidelines, specifically the Principles of Laboratory Animal Care (National Institutes of Health Publication 85–23, revised 1985).

### Microarray analysis

Total RNA, including both large and small RNAs, was extracted from mouse cortices using the mirVana PARIS kit (Ambion, Austin, TX, USA) according to the manufacturer’s instructions. The purity and the quality of the RNA samples were evaluated using a Nanodrop (Thermo Scientific, Hudson, NH, USA) and Bioanalyzer (Agilent, Santa Clara, CA, USA), and conformed to the required levels for microarray analysis. For the miRNA array, total RNA was labeled using a miRNA Complete Labeling and Hyb kit. For the gene expression array, total RNA was labeled using a Low Input Quick Amp Labeling Kit (Agilent). The RNA was hybridized to a Mouse miRNA Release 16.0 8×60 K array (Agilent) and a SurePrint G3 Mouse GE 8×60 K array (Agilent), respectively, according to the manufacturer’s protocols. GeneSpring GX (Agilent) and Ingenuity Pathway Analysis (IPA) software (Ingenuity Systems, Redwood City, CA, USA) were employed to process the output data from the microarrays.

### Quantitative real-time polymerase chain reaction (qRT-PCR)

According to manufacturer’s protocol, the expression levels of miRNAs and mRNAs were determined using TaqMan^TM^ MicroRNA Assays (Applied Biosystems, Foster City, CA, USA) and TaqMan^TM^ Gene Expression Assays (Applied Biosystems), respectively. The reverse transcription reaction was carried out with TaqMan^TM^ MicroRNA Reverse Transcription Kit (Applied Biosystems) and the High Capacity cDNA Reverse Transcription Kit (Applied Biosystems), respectively. The synthesized cDNA samples were amplified using TaqMan^TM^ Universal PCR Master Mix, no UNG (Applied Biosystems). qRT-PCR was performed using the 7300 Real-Time PCR System (Applied Biosystems). The Ct values of miRNA and mRNA were normalized using U6-snRNA and GAPDH, respectively, and evaluated using the 2^–ΔΔCt^ method.

### Isolation of primary murine neuronal cells

Primary murine neuronal cells (PMNCs) were isolated as follows. Briefly, embryonic brains were dissected and cortical tissues were isolated under sterile conditions. The dura mater and pia mater were removed carefully, followed by mincing of the cortical tissues. To produce a single cell suspension, 250 μL of trypsin (2.5%) and 250 μL of DNase I (2 mg/mL) were added to a 5 mL suspension of minced tissue in phosphate-buffered saline (PBS), and incubated for 20 min at 37°C. After removing the supernatant containing excessive membranes, (1) 150 μL of DNase I (2 mg/mL) and 7 mL medium for PMNCs were added to the cell pellets, and single cell suspensions were made by gentle pipetting. (2) The cell suspensions were incubated for 5 min by standing to separate cells in the supernatant from the tissue debris in the pellet. Then, step (1) and (2) were repeated, the supernatants were subsequently pooled in a new tube and centrifuged at 300 × *g* for 5 min at 4°C. The cells were then counted and seeded at 1 × 10^6^ cells/mL on 100 mm^2^ culture dishes pre-coated with poly-D-lysine.

### Cell culture and transfection of miRNA

PMNCs were maintained in Dulbecco’s modified Eagle medium/F12 (DMEM/F12; Invitrogen, Carlsbad, CA, USA) containing bovine albumin fraction V (1:75), 100 μg/mL apo-transferrin (Invitrogen), 5 μg/mL insulin from bovine pancreas (Sigma-Aldrich, St. Louis, MO, USA), 5.2 ng/mL sodium selenite (Sigma–Aldrich), 6.3 ng/mL progesterone (Sigma-Aldrich), and 16 μg/mL putrescine dihydrochloride (Sigma-Aldrich). The cells were cultured at 37°C in a humidified incubator with 5% CO_2_. The culture dishes and plates were pre-coated with poly-D-lysine (Sigma–Aldrich).

SH-SY5Y cells were cultured in DMEM/F12 medium (Gibco, Carlsbad, CA, USA) supplemented with 10% fetal bovine serum (FBS; Sigma–Aldrich) and 1% penicillin/streptomycin (Wako Pure Chemical Industries, Ltd., Osaka, Japan). The medium was changed to a serum- and antibiotic-free medium immediately before miRNA transfection. Primary neuronal cells and SH-SY5Y cells were transfected with miRNA mimics, Pre-miR miRNA precursors, or Negative Control #1 (NC) (Ambion, Austin, TX, USA) using Lipofectamine RNAiMAX transfection reagent (Invitrogen) according to the manufacturer’s protocol.

### Enzyme-linked immunosorbance assay (ELISA)

Levels of Aβ1–40 and Aβ1–42 secreted into conditioned medium were quantitated using a Human/Rat β Amyloid (40) ELISA Kit Wako II and Human/Rat β Amyloid (42) ELISA Kit v High-Sensitive (Wako), respectively. Briefly, PMNCs were plated in 6-well plates and, after 2 days of culturing, the serum was washed out using three changes of sterilized PBS. Serum-free medium was added to each well and, after 48 h, the incubation-conditioned medium was collected and diluted 1:5 for the Aβ1–40 assay and 1:2 for the Aβ1–42 assay, in a standard dilution buffer provided by the manufacturer.

### Preparation of oligomeric Aβ

Oligomeric Aβ1–42 (oAβ) was prepared as previously described [22]. Briefly, human Aβ1–42 (Peptide institute, Inc., Osaka, Japan) peptide was dissolved to 1 mM in 100% 1,1,1,3,3,3,-hexafluoro-2-propanol (HFIP) and dried down in a vacuum centrifugal evaporator. Then, the monomeric Aβ was resuspended to a 5 mM concentration in dimethyl sulfoxide (DMSO) and diluted to 100 μM in ice-cold cell culture medium DMEM-F12 (glutamine free) and left to oligomerize overnight at 4°C. Immediately after dilution to a final concentration of 2.5 μM with PBS, the oAβ peptide was flash-frozen in liquid nitrogen and stored at −20°C until use. The peptide solution was separated on a 5–20% Super Sep^TM^ Ace sodium dodecyl sulfate polyacrylamide (SDS-PAGE) gel (Wako), followed by Coomassie brilliant blue-staining to confirm the production of oAβ.

### Transfection of miRNA into mouse brain

The Accutarget miRNA mimic miR-200b/c (a mixture containing equal molar concentrations of miR-200b and miR-200c) or Negative control #1 (NC) (HPLC-grade; Bioneer, Daejon, Korea) was prepared for intracerebroventricular (i.c.v.) transfection into mouse brains, using Invivofectamine® 2.0 reagent (Invitrogen) in accordance with the manufacturer’s protocol. Briefly, 0.75 mg/mL miRNA in Invivofectamine®® solution was incubated for 30 min at 50°C, followed by dialysis using micro Float-A-Lyzer (molecular cut-off 8–10 kD; Spectrum Laboratories Inc., Rancho Domingo, CA, USA) in PBS (pH 7.4) for 2 h. The Invivofectamine® 2.0−miRNA duplex mixture was injected into the brain as described previously [23]. C57BL/6J mice were anesthetized with sodium pentobarbital (40 mg/kg, intraperitoneally [i.p.]) and a guide cannula (7 mm, 0.4-mm inner diameter, 0.5-mm outer diameter; Eicom, Kyoto, Japan) implanted into the right lateral ventricle (coordinates: −0.3 mm anteroposterior, 1.0 mm mediolateral from bregma, −2.5 mm dorsoventral from the skull). To seal the top of the guide cannula, a dummy cannula (0.3 mm in diameter; Eicom) was left in place throughout the experiment. One day after recovery from surgery, 21 mice were divided into two groups and mice in each group were treated with 2 μL vehicle (PBS) (n = 10) or oAβ (n = 11) through an infusion cannula (7.2 mm, 0.3 mm in diameter; Eicom) that was connected to a microsyringe by a polyethylene tube. In each group, an additional 3 μL of NC (vehicle: n = 5; oAβ: n = 5) or miR-200b/c (vehicle: n = 5; oAβ: n = 6)-Invivofectamine® duplex was co-injected with the vehicle or oAβ at a flow rate of 1 μL/min; mice were handled gently to minimize stress. Twenty hours after the infusion of oAβ and miRNA, mice were subjected to behavioral testing. Time spent on an injection of total 5 μL cocktail was about 5 minutes. The infusion was conducted daily during habituation and training (S1 Fig).

### Behavioral testing

The spatial learning impairment induced by oAβ was assessed using the Barnes maze, which has been described elsewhere [24, 25]. The apparatus consisted of a white circular disk (100-cm diameter, 75.5-cm high from the floor) with 12 holes (4 cm diameter) equally arranged like a clock face. One escape hole contacted the hole of the black acrylic escape box (13 × 17 × 7 cm) immediately below the disk, and the residual holes remained open. Each trial began with placing the mouse in a white acrylic cylinder (17-cm high, 11-cm diameter) in the center of the maze. After approximately 10 s, the cylinder was sunk below the surface of the maze remotely by the experimenter, which allowed mice to start at a random orientation. If the mouse did not enter the escape hole within 300 s, the experimenter gently guided it there. The mouse was left in the escape box for 60 s before being returned to its home cage. After each trial, the maze was rotated, and the maze and escape box were cleaned with 70% ethanol to prevent odor cues. The mice performed four trials per day for five consecutive days. The position of the escape hole remained constant for all mice over the training session. The mice could learn the position of the escape hole by using the objects hung in the experimental room as visual cues. The individuals that did not enter the escape hole for eight successive trials were excluded from this test. On the sixth day, the mice were subjected to a 300-s probe test on the maze in order to test their memory of the location of the escape hole, from which the escape box had been removed. During all trials as well as the probe test, the entire view of the maze was recorded using a CCD camera connected to a computer, and the data were analyzed using MatLab (Mathworks Inc., Natick, MA, USA) software, which allows automated tracking and analysis of escape paths.

### *In situ* hybridization

On the same day as the probe test finished, mice were anesthetized and perfused transcardially with cold 4% paraformaldehyde in PBS, pH 7.4. The brains were removed and immersed in 30% sucrose in PBS and embedded in optimum cutting temperature compound (Sakura Finetek Japan, Tokyo, Japan). The brain sections (10-μm thick) were cut on a cryostat, transferred to a glass slide, and kept at −20°C. To detect miR-200b expression, a 3′ and 5′-digitoxin (DIG)-labeled locked nucleic acid (LNA) probe was purchased from Exiqon (Woburn, MA, USA). *In situ* hybridization was performed as previously described, with slight modifications [26]. Briefly, the tissue on the slide was fixed in 4% paraformaldehyde (PFA) in PBS for 10 min, and rinsed in PBS for 3 min, three times. The slides were immersed in the acetylation solution (100 mM triethanolamine and 2.5 mM acetic anhydride/HCl, pH 8.0), which was prepared at the time of use, with stirring, for 10 min. After rinsing the slides in PBS for 5 min, samples were treated with 5 μg/mL Proteinase K (Roche Diagnostics, Mannheim, Germany) in PBS for 5 min. Then, the slides were washed in PBS for 20 min and prehybridized in a hybridization buffer (50% formamide, 5× Denhardt's solution, 5× standard saline citrate (SSC), 0.2 mg/mL yeast RNA (Sigma–Aldrich), 0.5 mg/mL salmon sperm DNA (Sigma–Aldrich), 2% blocking reagents) at room temperature (RT) for 4 h. The LNA DIG-labeled probe (20 nM) was prepared in hybridization buffer supplemented with 0.25% CHAPS and 0.1% Tween 20, and hybridization was performed at 60°C overnight. The slides were soaked in 5× SSC followed by 0.2× SSC at 60°C for 1 h and rinsed in B1 solution (0.1 M Tris pH 7.5, 0.15 M NaCl) at RT for 10 min. For immunostaining, the slides were pre-incubated in blocking solution (10% FBS, 0.05% Tween 20 in B1 solution) for 1 h and incubated using an anti-DIG-alkaline phosphatase antibody (1:2000; Roche Diagnostics) in the blocking solution at 4°C overnight. The slides were rinsed in the B1 solution and equilibrated in the B3 solution (0.1 M Tris pH 9.5, 0.1 M NaCl, 50 mM MgCl_2_). To detect alkaline phosphatase, the slides were incubated with 340 μg/mL nitroblue tetrazolium (NBT), 175 μg/mL 5-bromo-4-chloro-3-indolyl phosphate (BCIP), 2.4 mM levamisole, and 0.05% Tween 20 in B3 buffer for 2 h. The staining reaction was stopped by washing slides in PBS containing 0.1% Tween 20 for 10 min, three times. The slides were mounted using VectaMount (H-5501; Vector Laboratories, Burlingame, CA, USA).

### RNA-binding protein immunoprecipitation microarray (RIP-chip)

Argonuate 2 (AGO2) immunoprecipitation was conducted using an RIP-assay kit for miRNA (MBL, Nagoya, Japan) following the manufacturer’s instructions, with minor modifications. Briefly, an anti-EIF2C2/Ago2 monoclonal antibody (RN005M; MBL) was incubated with Dynabeads Protein G (Life Technologies, AS, Oslo, Sweden) at 4°C for 30 min to prepare antibody-immobilized beads. A mouse IgG1 isotype control (M075-3; MBL) was used as a negative control. The SH-SY5Y cells transfected with 5 nM pre-miR-200b, -200c, or NC (Ambion) for 24 h were lysed with 500 μL of lysis buffer. The lysate was incubated with Dynabeads Protein G without antibody at 4°C for 30 min to reduce non-specific adsorption. Then, the cell lysate was transferred into a tube containing antibody-immobilized Dynabeads Protein G, and incubated for 4 h at 4°C. The complex was washed four times with wash buffer followed by large and small RNA isolation. The total RNA was labeled using a Low Input Quick Amp Labeling Kit (Agilent). The RNA was hybridized to a SurePrint G3 Human GE 8×60 K array (Agilent), according to the manufacturer’s protocol. GeneSpring GX (Agilent) was employed to process the output data.

### Co-transfection and luciferase reporter assay

The SH-SY5Y cells were plated in 24-well plates. After 48 h, using Lipofectamine 3000 (Invitrogen), the cells were co-transfected with 25 nM pre-miR-200b, -200c, or NC (Ambion) and with 0.5 μg of pEZX-MT01 plasmid (GeneCopoeia, Rockville, MD, USA) expressing the 3'-UTR target sequence clone, ribosomal protein S6 kinase B1 (S6K1; cat no. HmiT066134-MT01), JUN (cat no. HmiT009853-MT01), serine/arginine-rich splicing factor 1 (SRSF1; 11–2495 bp and 2384–4826 bp of separated sequence clone; SRSF1-a; cat no. HmiT016880a-MT01, and SRSF1-b; cat no. HmiT016880b-MT01), respectively, or a negative control clone (cat no. CmiT000001-MT01). For zinc finger E-box-binding homeobox 1 (ZEB1; cat no. HP215380), the plasmid expressing the 3'-UTR target sequence clone was purchased from OriGene Technologies, Inc. (Rockville, MD, USA). The dual luciferase activity of cells after 24 h of transfection was measured using a Luc-Pair™ miR Luciferase Assay kit (GeneCopoeia, cat. no. LPFR-M030) according to the manufacturer's protocol. Firefly luciferase activity was normalized to Renilla luciferase activity in the same well.

### Western blot

SH-SY5Y cells transfected with 25 nM pre-miR-200b, -200c, or NC (Ambion) for 48 h were harvested with radioimmunoprecipitation (RIPA) buffer (Sigma–Aldrich). The cell lysates were separated on 5–20% Super Sep^TM^ Ace SDS-PAGE gels (Wako) and transferred to polyvinylidene fluoride membranes (GE HealthCare, Little Chalfont, UK). Blots were blocked in 5% skimmed milk at RT for 30 min and incubated at 4°C overnight in Canget Signal solution 1 (TOYOBO, Osaka, Japan) containing one of the following: anti-Actin (C-11; Santa Cruz Biotechnology, Santa Cruz, CA, USA; 1:5000), anti-ZEB1 (E20; Santa Cruz Biotechnology; 1:1000), anti-S6K1 (E343; Abcam, Cambridge, MA, USA; 1:5000), anti-SRSF1 (12929-2-AP; Proteintech, Chicago, IL, USA; 1:5000), anti-IRS1 (1:500), anti-phospho-IRS1 (S302/307; 1:1500), anti-phospho-IRS1 (S1097/1101; 1:1500), anti-phospho-IRS1 (S307/312; 1:3000), anti-phospho-IRS1 (S636/639; 1:1500), or anti-JUN (60A8; 1:2500) antibodies (the latter all purchased from Cell Signaling Technologies; Beverly, MA, USA). Following washing in Tris-buffered saline with 0.5% Tween 20 (TBS-T), the membranes were incubated at RT for 2 h with rabbit anti-goat IgG-HRP or donkey anti-rabbit IgG-HRP in Canget Signal solution 2 (TOYOBO). Then, immunoreactive bands were visualized using Immunostar LD (Wako), and luminescent images were analyzed using a LAS-4000 imager (Fujifilm, Tokyo, Japan).

### Statistical analysis

All data are expressed as mean ± SEM. Statistical analysis was performed using StatFlex version 6 software (Artech Co. Ltd. Osaka, Japan). In the microarray data and the experiment *in vitro*, the means of two groups were tested by an unpaired Student’s *t* test. In the experiment *in vivo*, a 2-way repeated measures ANOVA was used to compare the escape latency and treatment groups in 5 days of training. A chi-square test was applied to compare the occupation rate and the value of chance level. A value of *P* < 0.05 was considered to be statistically significant.

## Results

### MiRNA and mRNA expression profile of the Tg2576 mouse brain

**Fig 1:**
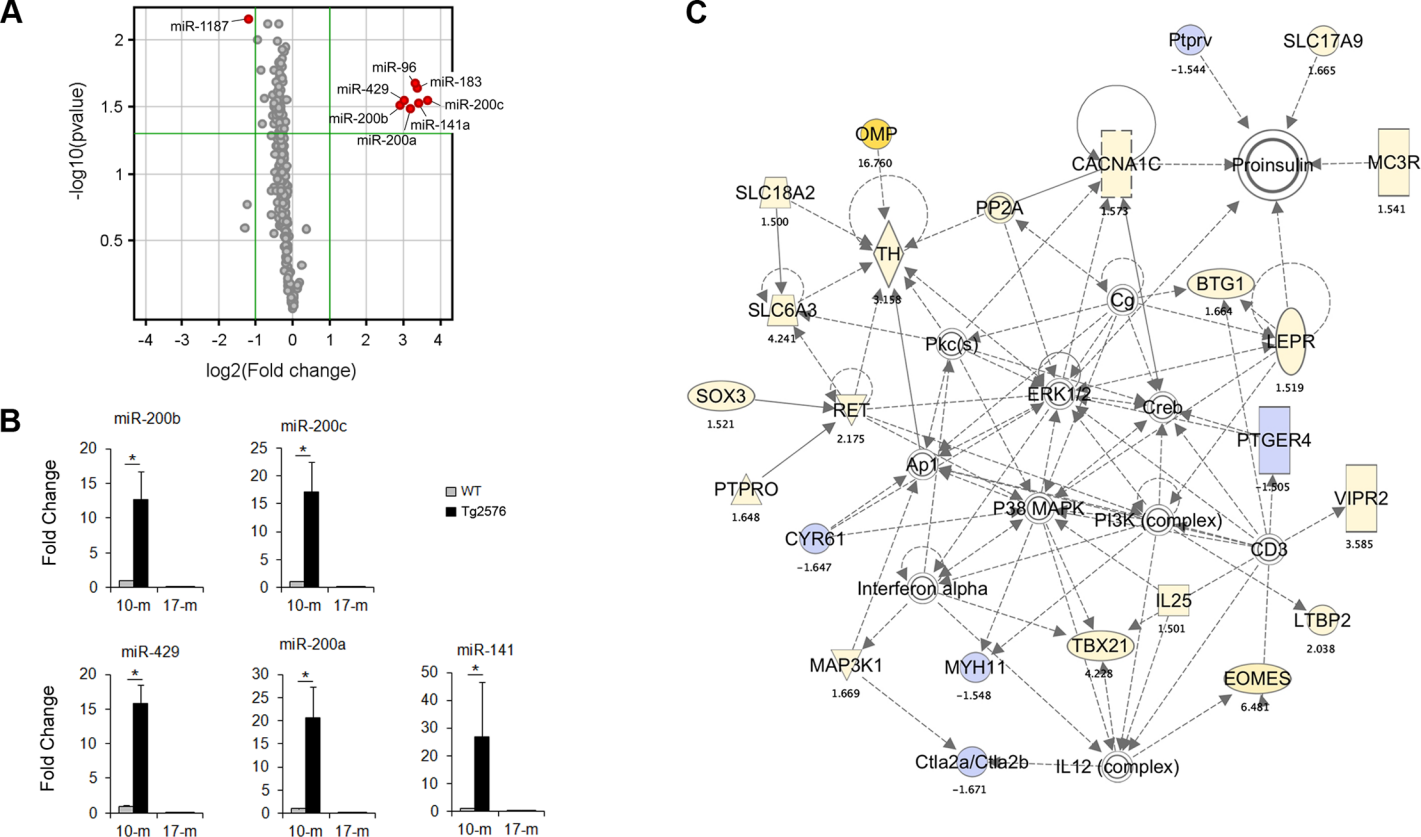
The members of the miR-200 family and related differentially expressed mRNA network are implicated in insulin signaling in the cortex of 10-month-old Tg2576 mice. (A) Volcano plot showing microarray data of significantly upregulated miRNAs in the cortices of 10-month-old (10 mo.) Tg2576 mice as compared to wild-type (WT) mice. The threshold values for fold change were set at > 2 or < 0.5 (outside the vertical lines) and P value < 0.05 (above the horizontal line); red points in the plots represent the significant differentially expressed miRNA. The miRNAs that were upregulated were members of the miR-200 and miR-183 families. (B) qRT-PCR was used to validate the upregulation of miR-200 family members using TaqMan assays. The upregulation of members of this miRNA family was confined to the 10 mo. period, a phase during which Aβ accumulation increases. (C) The top network of differentially expressed mRNA as identified by IPA analysis of the microarray data. Up- and down- regulated genes are colored in yellow and blue, respectively. Solid and dashed arrows indicate direct and indirect connections, respectively. Subscripted numbers indicate fold change. The network involves components of the insulin signaling pathway.

The miR-200 family was upregulated in the cortices of 10-month-old Tg2576 mice as compared with age-matched WT mice in the microarray analysis. While some downregulated miRNAs were observed, we focused on the significantly upregulated miR-200 family members (miR-141, -200a, -200b, -200c, and -429) and the miR-183 family members (miR-96, -182, and -183), which had higher expression levels in 10-month-old Tg2576 mice (fold-change > 2.0, unpaired Student’s *t*-test; *p* < 0.05; Fig 1A).

We used 10-month-old and 17-month-old mice to confirm the miRNA response to the early period of Aβ accumulation in the Tg2576 mouse brain, since Tg2576 mice show increased Aβ levels from 9–10 months of age and show significant cognitive memory loss around 15–18 months of age [27]. With regard to the comparison between the cortices of 17-month-old WT vs. Tg2576 mice, only miR-1187 was significantly decreased. To validate the upregulation of the above family members in the cortices of 10-month-old mice, the expression levels were quantified using qRT-PCR. All miRNAs showed marked increases in miRNA expression levels in 10-month-old, but not in 17-month-old mice, which was consistent with the microarray data (Fig 1B). These results suggest that some miRNAs may respond to the start of Aβ accumulation, and have the potential to play key roles in the early stages of AD by controlling target gene expression.

To investigate the gene expression network involved in this model, we conducted a biological pathway analysis on genes that were differentially expressed (fold-change > 1.5, unpaired Student’s *t*-test; *p* < 0.05) in the cortices of 10-month-old Tg2576 mice as compared to WT mice. The top network identified by IPA software included proinsulin, extracellular signal-regulated kinase 1/2 (ERK1/2), phosphoinositide 3 kinase (PI3K), P38 mitogen-activated protein kinase (MAPK), and cAMP response element binding protein (Creb), amongst others (see Fig 1C). Thus, the members of the miRNA family identified appeared to play a role in insulin signaling.

### Effect of miR-200b and miR-200c on Aβ-induced toxicity *in vitro*

**Fig 2:**
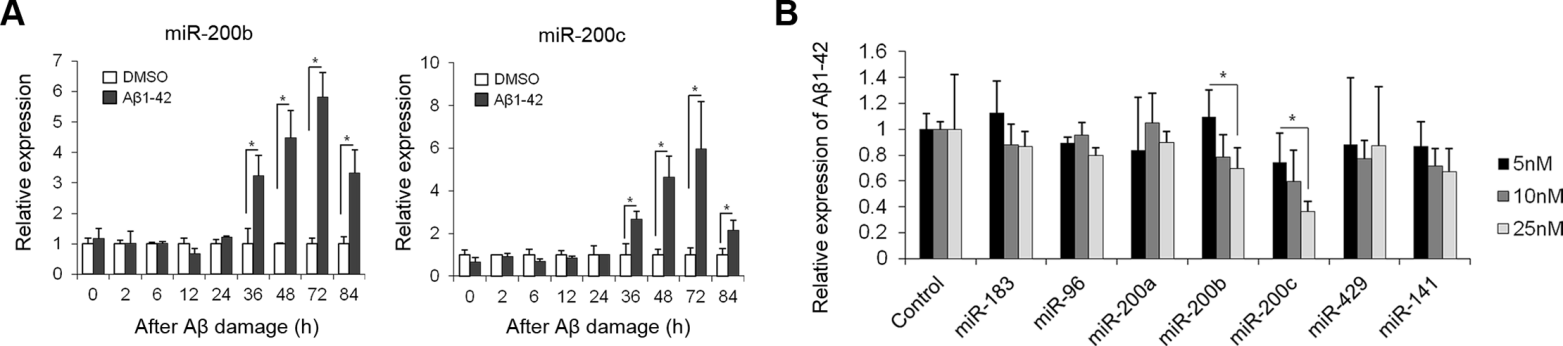
Suppressive effect of miR-200b and miR-200c on Aβ generation *in vitro*. (A) Aβ1–42 dramatically induced the expression of miR-200b and miR-200c in PMNCs. PMNCs were exposed to DMSO as control or 5 μM Aβ1–42 in DMSO. The results for miR-200b (left) and miR-200c (right) are shown as expression of miRNA in the treated sample relative to that of the DMSO treated control sample at each time point. *p < 0.05, n = 6. Data are expressed as mean ± standard error of the mean (SEM). (B) miR-200b and miR-200c attenuate Aβ secretion. Cells were treated with miRNAs at different concentrations (5 nM, 10 nM, and 25 nM). The Aβ1–42 secreted into the conditioned medium from neuronal cells was measured by ELISA. The results shown are relative to the levels in control transfected cells for each miRNA concentration. *p < 0.05, n = 3. Data are expressed as mean ± SEM.

To verify whether expression of miR-200b and miR-200c are altered in response to the neuronal damage induced by Aβ1–42, we treated PMNCs with Aβ1–42 peptides and examined miRNA expression levels using qRT-PCR. The miRNA expression in the treated cells was compared to that in cells treated with DMSO as a control. Both miR-200b and miR-200c expression levels in PMNCs were markedly increased by Aβ1–42 stimulation after 36 h of treatment, and the peak in the elevation of miRNA expression was observed at 72 h, with a six-fold increase being found (Fig 2A). Since Aβ is secreted into the culture medium of PMNCs, conditioned media from PMNCs were obtained at 24-h post-transfection to measure secreted Aβ1–42 levels. Surprisingly, Aβ1–42 secretion was markedly suppressed in miR-200b and miR-200c transfected PMNCs, and the impairment of Aβ secretion in the conditioned medium was dependent on the miRNA concentrations used (Fig 2B). Taken together, we speculate that miR-200b and miR-200c expression in neuronal cells has the potential to suppress the cytotoxic damage caused by Aβ, especially Aβ1–42, in the early stages of AD by regulating Aβ secretion.

### Effects of miR-200b and miR-200c on Aβ-induced toxicity *in vivo*

**Fig 3:**
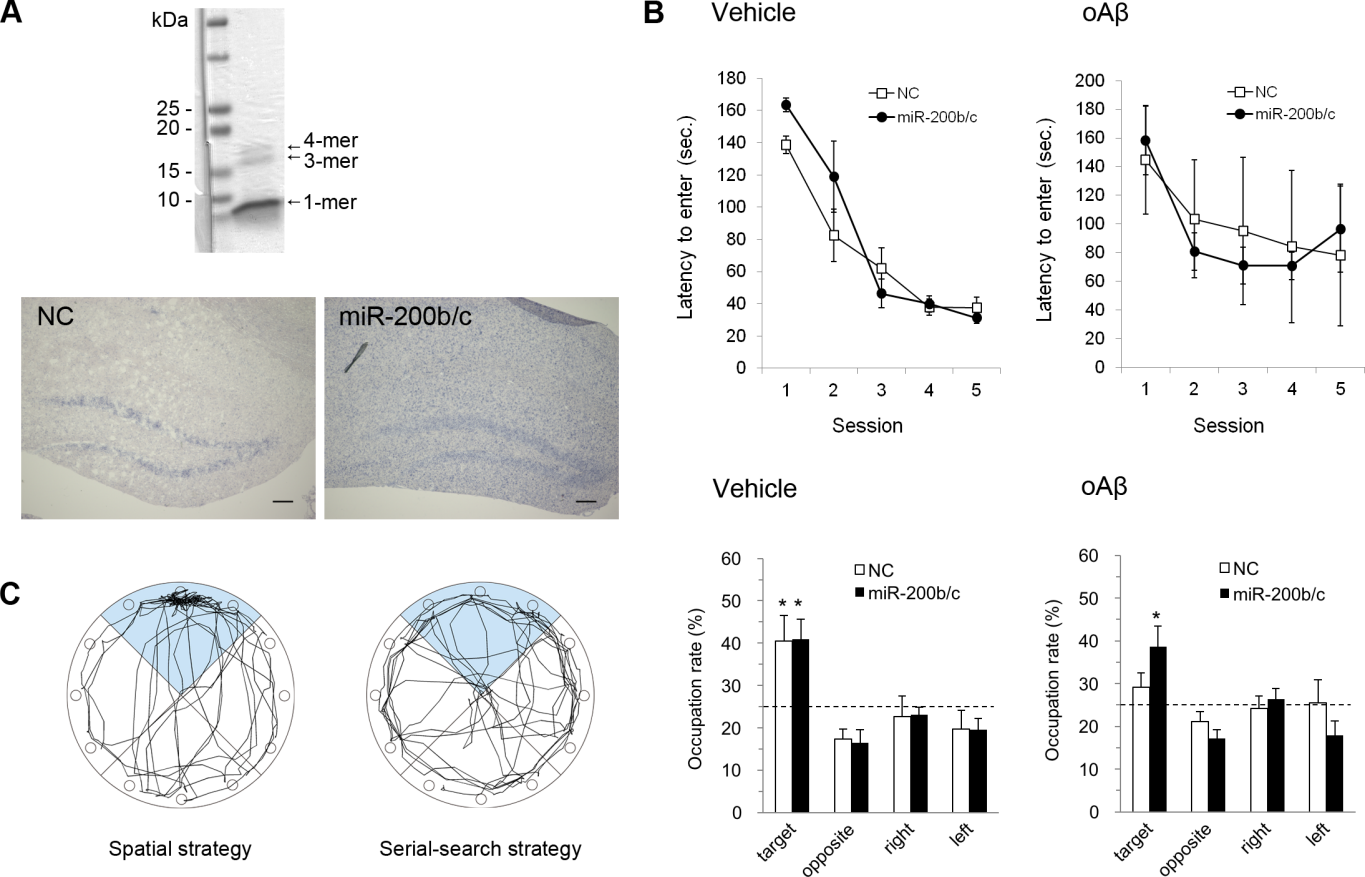
Defensive effect of miR-200b/c on oligomeric Aβ-induced learning impairments *in vivo*. (A) Upper image: Coomassie brilliant blue-stained oAβ following separation using SDS-PAGE gels. Both 4-mer and 3-mer Aβ were reliably observed. Lower images: *In situ* hybridization image showing detection of the miR-200b probe in the hippocampi of NC (left) and miR-200b/c (right) i.c.v.-infused mice. The hippocampi from the mice overexpressing miR-200b/c exhibited higher intensity digitoxin labeling than NC hippocampi. Scale bar, 100 μm. (B) Upper graphs: the latency to enter a goal hole during the training sessions over 5 days in the vehicle (left) and oAβ (right) groups. In both groups, NC- and miR-200b/c-treated mice showed improved performance with the number of sessions (two-way ANOVA, *F* (4, 40) = 50.0, *p* < 0.00001). Lower graphs: percentage of time spent in each quadrant for the vehicle- (left) and oAβ- (right) treated groups during the probe test. The results of the probe test showed that spatial learning in miR-200b/c-infused mice was not influenced by oAβ (Chi-square test, *p < 0.01). (C) Representative path tracing of mice using a spatial strategy (left) and a serial-search strategy (right).

In order to evaluate the inhibitory effect of miR-200b/c on Aβ-induced toxicity *in vivo*, the spatial memory of oAβ injected mice was tested using the Barnes maze. The i.c.v. injection of oAβ has been shown to induce impairments in spatial memory, which are reversible [24, 25]. Before treatment, the oAβ solution was confirmed to contain monomers, trimers, and tetramers using SDS-PAGE (Fig 3A). We infused the miRNA-Invivofectamine® duplex i.c.v. to transfect miR-200b/c, and verified their overexpression in the hippocampus using *in situ* hybridization (Fig 3A). For the successive 5 days of training sessions, a two-way ANOVA revealed that there was no NC or miR-200b/c treatment effect (*F* (1, 9) = 0.94, *p* = 0.36) or treatment-by-session interaction (*F* (4, 32) = 2.38, *p* = 0.072) for the vehicle group. There was no effect on the latency time to enter the goal (Fig 3B). NC- and miR-200b/c-treated mice showed improved performance with increasing session number, and there was a significant effect on the latency time (*F* (4, 40) = 50.0, *p* < 0.00001). Likewise, in the training session for the oAβ-injected group, there was no effect of NC or miR-200b/c treatment (*F* (1, 10) = 0.018, *p* = 0.90) or treatment-by-session interaction (*F* (4, 36) = 0.70, *p* = 0.60) on the latency time (Fig 3B). Although the latency time varied widely in individuals in the oAβ group, session had a significant effect (*F* (4, 44) = 6.59, *p* < 0.0004).

The chi-square test results for the 300-s probe test clarified that both NC- and miR-200b/c-treated mice in the vehicle group stayed in the target quadrant significantly longer than by chance, i.e., 25% (NC: *p* < 0.005; 200b/c: *p* < 0.005). In the oAβ group, miR-200b/c-treated mice spent significantly more time in the target quadrant than by chance (*p* < 0.01), although NC-treated mice spent a similar amount of time in all quadrants (*p* = 0.72). If the mouse appropriately memorized the goal location using spatial cues, their path tracing during the probe test converged onto the target quadrant, as indicated in Fig 3C. On the other hand, NC-treated mice in the oAβ group showed path tracing in the perimeter of the maze that would lead to incidentally finding the goal by moving from one hole to the next hole, which implies that they used a serial-search strategy.

### Identification of miR-200b and miR-200c targets

To elucidate the miR-200b and miR-200c signaling pathways involved in Aβ-induced toxicity, we explored their direct targets using RIP-Chip. The RIP-Chip assay can biochemically identify target mRNAs that are co-immunoprecipitated with a ribonucleoprotein, AGO2, followed by microarray analysis. A total of 171 and 204 genes were immunoprecipitated as candidate targets of miR-200b and miR-200c, respectively (fold-change > 2.0, *t*-test; *p* < 0.05; S1 Table). The above-mentioned network consisted of differentially expressed genes in 10-month-old Tg2576 mouse cortex presenting highly expression of miR-200 family (Fig 1C). This could include downstream genes of target genes post-transcriptionally inhibited by miR-200b or miR-200c. Hence, we attempted to establish a network between the direct target genes obtained using RIP-Chip and the downstream genes consisting of the top network of differentially expressed genes in Tg2576 mouse. As a result, the IPA analysis enabled us to narrow the candidate targets down to 17 genes that were connected with downstream genes (S2 Fig). Moreover, taking *in silico* (TargetScan: http://www.targetscan.org, DIANA-microT-CDS: http://diana.imis.athena-innovation.gr/DianaTools/index.php?r=microT_CDS) predictions into consideration (S1 Table), the selected targets were validated by luciferase assays, qRT-PCR, and western blot analyses. We used ZEB1, which has been shown to be a target for miR-200b and miR-200c, as a positive control in these experiments. We also selected SRSF1 which was top-ranked among immunoprecipitated genes by RIP-Chip and has been reported to regulate S6K1 splicing.

**Fig 4:**
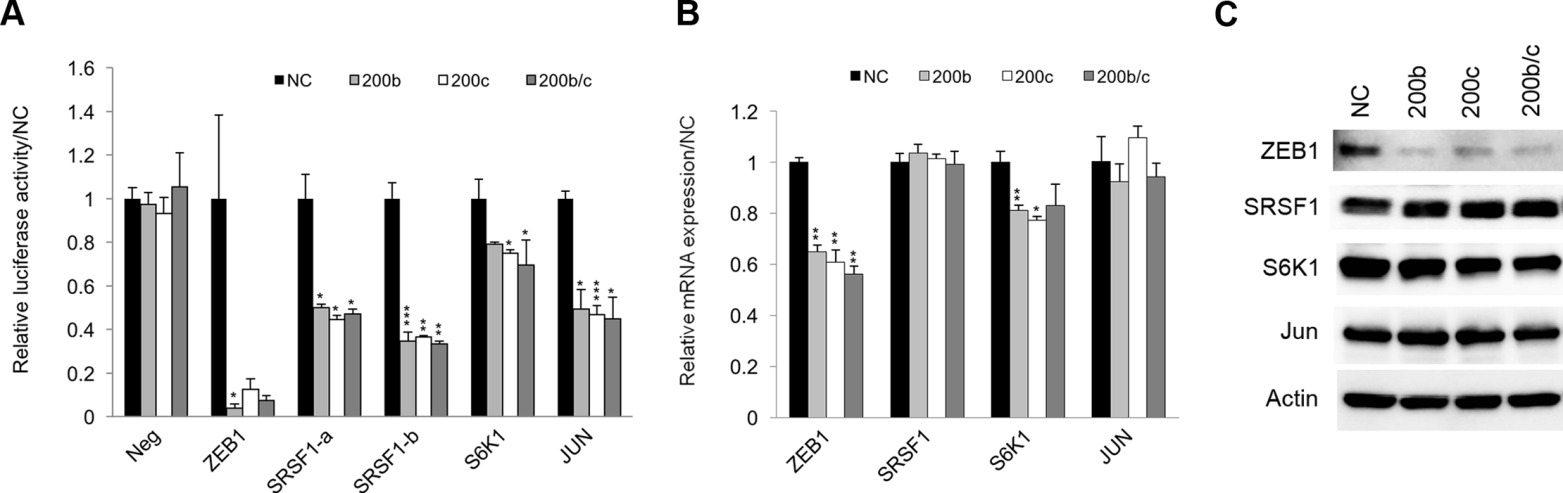
Target validation of SRSF1, JUN, and S6K1 using a luciferase reporter assay, qRT-PCR, and western blot analysis. (A) A luciferase reporter assay was performed in SH-SY5Y cells 48 h after co-transfection with the miRNAs and each luciferase reporter plasmid expressing the 3′-UTR target sequence clone. The relative luciferase activities of cells containing a target 3′-UTR were calculated as compared with the value in NC cells. Neg: negative control clone. *p < 0.05, **p < 0.01, ***p < 0.001, n = 3 (B) qRT-PCR was performed on cDNA from SH-SY5Y cells 24 h after transfection with miRNAs. The relative expressions of target mRNAs were calculated in comparison with the levels in the NC cells. *p < 0.05, **p < 0.01, ***p < 0.001, n = 3 (C) Western blot analysis was performed on protein from SH-SY5Y cells 48 h after transfection with miRNAs. Equal protein loading was determined by assessing the levels of actin.

The luciferase assay was performed for ZEB1, SRSF1, S6K1, and JUN 3'-UTR, as they include target sites with higher probabilities of preferential conservation in the TargetScan database. The activities of all of these were significantly reduced by miR-200b and/or miR-200c transfection (Fig 4A). Although their diminished levels did not correspond with the number of target sites or scores predicted from the 3'-UTR sequence *in silico*, all candidate genes were demonstrated to have direct target sites for miR-200b or miR-200c.

The mRNA expression levels were also evaluated by qRT-PCR, since miRNAs reduce transcript levels in part due to mRNA cleavage. Predictably, the inhibitory effects on each gene were low, although significant differences were observed in ZEB1 and S6K1 levels in SH-SY5Y cells transfected with miR-200b and miR-200c compared to NC (Fig 4B). Western blot data showed that ZEB1 was clearly downregulated in miR-200b and miR-200c transfected SH-SY5Y cells (Fig 4C). The levels of S6K1 were somewhat decreased in miR-200c-transfected cells. No other targets were differentially expressed in miRNA-transfected cells.

### S6K1-dependent serine phosphorylation of IRS-1

**Fig 5:**
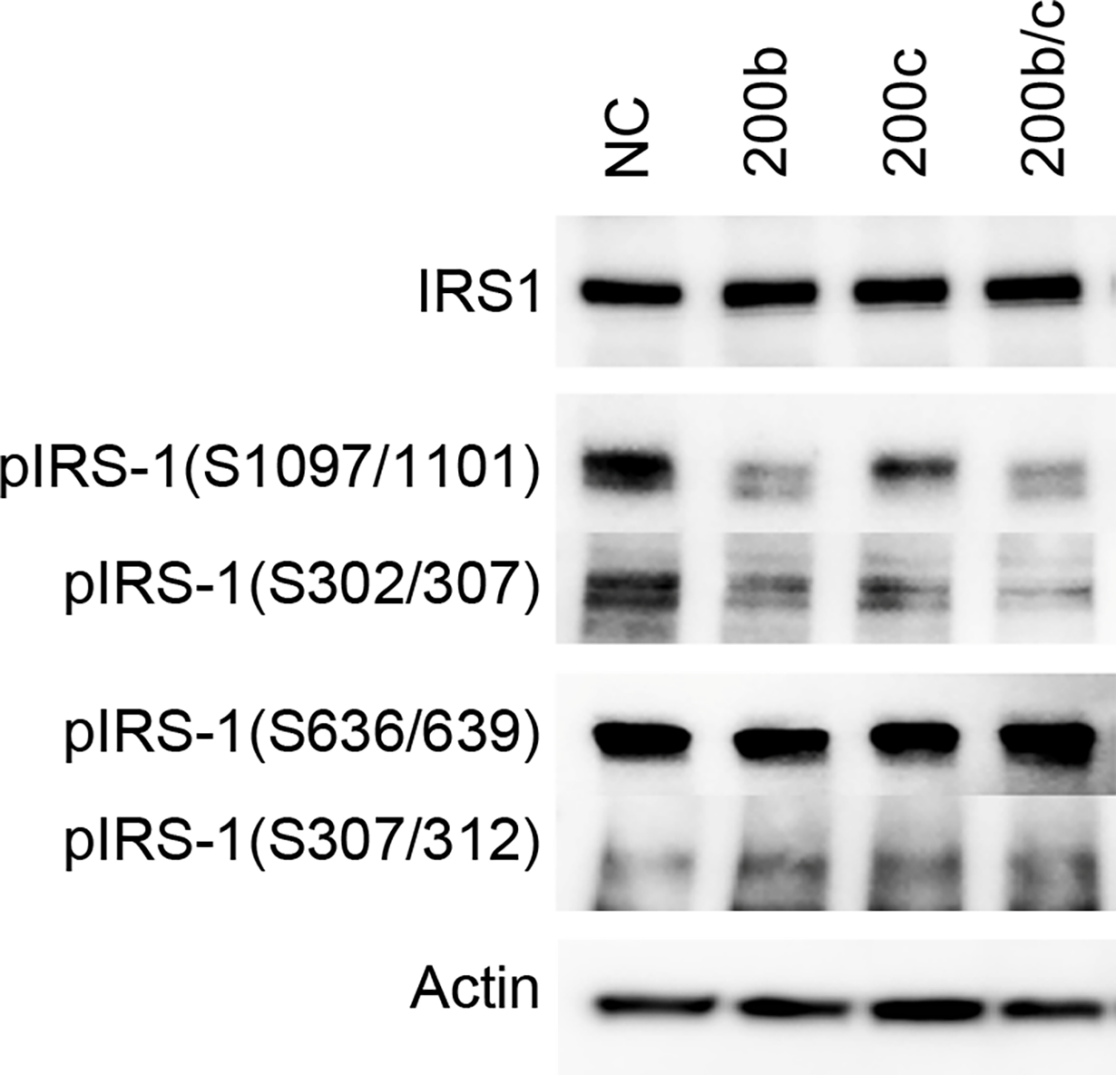
S6K1-dependent serine phosphorylation of IRS1 in SH-SY5Y cells transfected with miR-200b and miR-200c. Western blot analysis for anti-phospho IRS-1 on serine (S) 302/307, S307/312, S636/639, and S1097/1101 revealed that downstream regulation of S6K1 was inhibited by miR-200b and miR-200c. The reduction in S302/307 and S1097/1101 phosphorylation suggests that S6K1 is a target of miR-200b and/or miR-200c. Equal protein loading was determined by analyzing actin levels.

Given that S6K1 is a target of miR-200b and/or miR-200c, we evaluated its downstream regulation. Mechanistically, S6K1 inactivates IRS-1 through phosphorylation of the serine (S) residues 307 and 1101, which leads to negative feedback to the PI3K/mTOR pathway. Increased serine phosphorylation of IRS-1 induces insulin resistance, suggestive of an association with AD pathogenesis. We postulated that miR-200b and/or miR-200c would inhibit S6K1, which would result in a reduction in the insulin resistance induced by oAβ. Western blot images showed that S6K1-dependent phosphorylation of IRS-1 on S307 and S1101, but not IRS-1 itself, were decreased in miR-200b- and miR-200c-transfected SH-SY5Y cells (Fig 5). On the other hand, the other residues, such as S312 and S636/639, were phosphorylated to the same degree. This finding suggests that miR-200c in part contributes to the promotion of insulin signaling through the PI3K/mTOR pathway.

## Discussion

Aβ peptide, derived by processing of APP by BACE1, is implicated in AD pathogenesis [3, 4, 28] and causes cytotoxic damage to neuronal cells, leading to memory loss [29, 30]. Therefore, miRNAs directly targeting APP and BACE1 are of interest as potential therapeutic molecules for both AD and AD mouse models [31–33]. In this study, we showed that miR-200 family members were upregulated in the cortices of 10-month-old Tg2576 mice, which might bring about compensative effects in relation to Aβ-induced toxicity. The marked upregulation of members of the miR-200 family was confined to the phase of increasing Aβ deposition, and disappeared in the plateau phase (17-month-old mice).

Previously, in 7-month-old APP23 mouse brains, it has been demonstrated that Aβ accumulation results in dysregulation of several miRNAs [34]. Another study reported that downregulation of miR-29a induces a compensatory response against neurodegeneration in the AD brain [35]. Thus, miRNA profile changes may reflect results and the subsequent feedback responses to pathological events. In this study, we investigated the function of the miR-200 family, the upregulation of which may represent an innate defense response rather than being the result of Aβ-induced toxicity. First, using *in vitro* models, we successfully reproduced the Aβ-induced upregulation of miR-200b and -200c in the media of cultured neurons. Moreover, transient transfection of neurons with miR-200b/c in particular diminished the secretion of Aβ into the conditioned media. These findings suggest that miR-200b and miR-200c have defensive roles against Aβ-induced toxicity.

Therapeutic miRNAs have been discovered and their effectiveness has been validated *in vivo*, predominantly in cancer research [20, 36, 37]. Although nanoparticles and exosomes can successfully delivery miRNAs into the brain [38], there exists no report to evaluate cognitive improvement using therapeutic miRNAs.

We used a non-viral transfection reagent, Invivofectamine®, to transfect miR-200b/c in mice *in vivo*, and examined whether these miRNAs could palliate spatial memory impairments induced by oAβ by using the Barnes maze test. Treatment with oAβ can reversibly disrupt spatial memory [24, 25], which is thought to be a result of synapse loss and inhibition of hippocampal-dependent long-term potentiation [39–41]. Mice preferentially use a spatial strategy, in so far as they could use distal room cues to learn the target location [42]. Among the mice that were injected i.c.v. with oAβ, the mice expressing NC switched from the spatial strategy to a serial-search strategy owing to spatial memory impairments. On the other hand, the mice overexpressing miR-200b/c in their hippocampi maintained the ability to memorize the spatial location. Thus, miR-200b/c may have defensive effect against Aβ-induced toxicity *in vivo*.

To gain further understanding of the defensive roles of these miRNAs in the brain, we employed a RIP-Chip assay that enabled us to find direct target mRNAs bound with miRNAs to AGO2 [43, 44]. Many tools are available to predict miRNA targets *in silico*, although they do not permit biologically meaningful identification of targets in the intended cells, tissues, and diseases. Growing evidence indicates that the miR-200 family is involved in cancer cell migration via the targeting of ZEB1 and ZEB2 [45–48]. Although neurological reports for the miRNA-200 family are few, one neurological study has shown that members of the miR-200 family contribute to olfactory neurogenesis [49], and a microarray study showed that miR-200c is differentially expressed in AD brains [50].

Our RIP-Chip assay results did not include BACE1, which is responsible for Aβ production. This reflects the poor predictive ability of *in silico* tools. We approached the target search by focusing on mRNAs involved in the insulin signaling pathway. Intriguingly, miR-8 in flies (homologous to miR-200 in mammals) has been revealed to target FOG2, which binds to a subunit of PI3K and activates insulin signaling [51]. However, in the present study, FOG2 (homologous to Zfpm2 in mouse) was not identified as a candidate gene in the RIP-assay, and it is expressed at low levels in the mouse brain (data not shown). Here, we demonstrated that several candidates from the integrated RIP-Chip and differentially expressed mRNA analysis in Tg2576 mouse brain. Based on the results from the gene expression assay and protein assays, S6K1 was a plausible target for miR-200b and/or -200c. However, considering that the result of luciferase assay was not prominent, S6K1 might be indirectly repressed by unidentified targets. Further study is required to elucidate overall mechanisms including other direct targets.

S6K1 is known to be a downstream effector of mTOR, and to induce insulin resistance via phosphorylation of serine residues of IRS1 as a feedback response [52]. Hyperphosphorylated serine residues of IRS-1 have been reported in the hippocampi of AD mouse models and monkeys injected i.c.v. with oAβ [53]. We observed that S6K1-dependent phosphorylation of IRS1 on S307 and S1101 was diminished in SH-SY5Y cells treated with miR-200b and miR-200c.

The oAβ leads to additional generation of oAβ resulted from abnormal autophagosome accumulation via inhibition of mTOR pathway [17]. The findings from our *in vitro* study suggest that miR-200b and/or miR-200c targeting of S6K1 could suppress Aβ generation through amelioration of insulin resistance. MiR-200b and/or miR-200c may play a defensive role with regard to intracellular Aβ production, as well as extracellular Aβ-induced toxicity by promoting insulin signaling. Felice and coworkers have demonstrated that insulin modulates levels of Aβ and protects against the detrimental effects of oAβ on synapses [13]. Our *in vivo* finding showed that miR-200b/c relieved oAβ-induced impairment of spatial memory. The therapeutic potential might be explained by further study to demonstrate protecting synapses from extracellular oAβ in miR-200b/c-infused models.

In conclusion, miR-200b/c-targeted inhibition of S6K1 expression might contribute to a reduction in Aβ secretion and/or Aβ–induced spatial memory impairment by promoting activation of the insulin signaling pathway in AD models.

## Supporting information

S1 Fig Experimental protocol used in behavioral test. (TIF)

S2 Fig A combined network of direct target genes resulting from the RIP-Chip assay upstream and the top network of differentially expressed mRNAs in the Tg2576 mouse brain downstream, generated with IPA software. (PNG)

S1 Table The list of immunoprecipitated (IP) genes were two-fold greater in SH-SY5Y cells transfected with miR-200b or miR-200c than in NC cells (t-test; *p* < 0.05). (XLSX)
