# Supplementary figures and images for: Defensive effect of microRNA-200b/c against amyloid-beta peptide-induced toxicity in Alzheimer's disease models
Source: PLoS One. 2018 May 8;13(5):e0196929. doi: 10.1371/journal.pone.0196929 (PMC5940223; doi:10.1371/journal.pone.0196929)

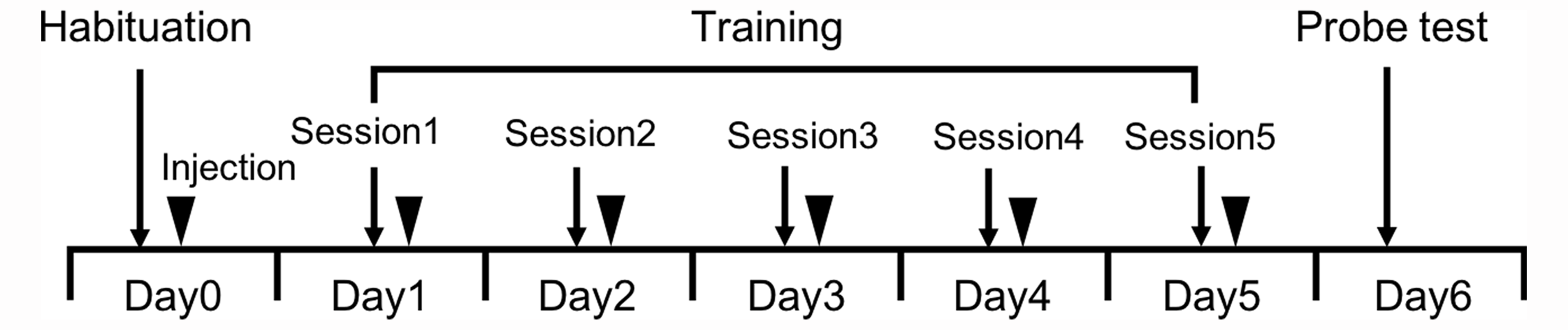

Supplement: S1 Fig — (TIF) [file pone.0196929.s001.tif]

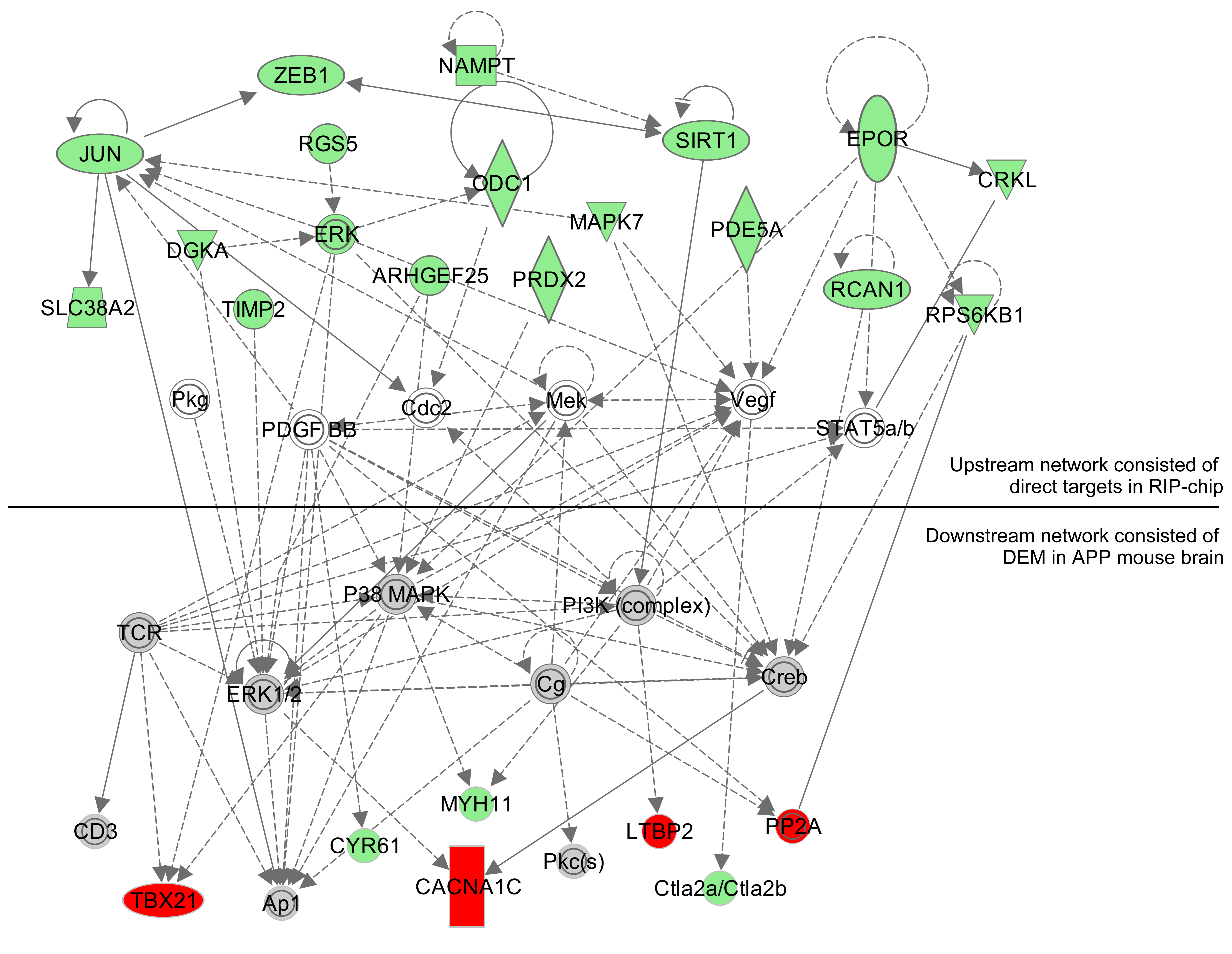

Supplement: S2 Fig — (PNG) [file pone.0196929.s002.png]
